# Validity of SC-StepRx pedometer-derived moderate and vigorous physical activity during treadmill walking and running in a heterogeneous sample of children and youth

**DOI:** 10.1186/1471-2458-14-519

**Published:** 2014-05-28

**Authors:** Travis John Saunders, Casey Ellen Gray, Michael Marc Borghese, Allison McFarlane, Afekwo Mbonu, Zachary Michael Ferraro, Mark Stephen Tremblay

**Affiliations:** 1 Healthy Active Living and Obesity Research Group, Children’s Hospital of Eastern Ontario Research Institute, 401 Smyth Road, Ottawa, Ontario, Canada; 2 School of Human Kinetics, University of Ottawa, 125 University Private, Ottawa, Ontario, Canada; 3 Department of Health Sciences, Lakehead University, 955 Oliver Road, Thunder Bay, Ontario, Canada

**Keywords:** Physical activity, Locomotion, Running, Walking, Activity monitors

## Abstract

**Background:**

The purpose of this study was to determine the validity of the SC-StepRx pedometer to assess moderate and vigorous physical activity during treadmill walking and running in a heterogeneous sample of children and youth aged 10-17 years.

**Methods:**

Physical activity intensity assessed via indirect calorimetry served as the criterion standard. A convenience sample of 40 participants (20 boys, 20 girls) wore 6 SC-StepRx pedometers, 2 ActiGraph GT3X accelerometers, 2 Actical accelerometers, 1 Walk4Life MVP pedometer and 1 NL-1000 pedometer while walking/running at speeds approximating 2, 3, 4, 6 and 7 METs. Associations between indirect calorimetry and each activity monitor were assessed using linear regression analyses in SAS 9.2.

**Results:**

Estimates of moderate and vigorous physical activity from all monitors were significantly associated with the criterion standard of indirect calorimetry. The strongest associations with the criterion measure were observed for the SC-StepRx with moderate/vigorous thresholds of 110/130 steps•minute^−1^, and the NL-1000 (R^2^ = 0.82, *p* <0.05). The SC-StepRx with moderate/vigorous thresholds of 110/130 steps•minute^−1^ also exhibited the highest combined sensitivity (92.9%) and specificity (96.5%) for correctly identifying a bout of moderate-to-vigorous physical activity.

**Conclusions:**

This study demonstrates that the SC-StepRx pedometer is a valid tool for the measurement of moderate and vigorous physical activity in children and youth.

## Background

High levels of physical activity are positively associated with both physical and mental health in children (aged < 12 years [1]) and youth (aged 12–17 years [1]) [2]. Pedometers, which measure the number of steps taken by an individual, are a common tool for the assessment of physical activity in the pediatric age group [3-5]. However, while traditional pedometers are able to provide an estimate of a child’s global physical activity level (e.g. steps•day^−1^), they are unable to determine whether that activity was performed at a light, moderate, or vigorous intensity. This is a major shortcoming, as health outcomes are more closely associated with moderate (MPA) and vigorous (VPA) physical activity than with light physical activity in pediatric populations [2]. Further, without the ability to assess MPA and VPA, pedometers have thus far been unable to determine whether a child meets Canada’s Physical Activity Guidelines [1].

To overcome this limitation, several companies have introduced pedometers that are capable of recording time spent engaging in MPA, VPA, or combined moderate and vigorous physical activity (MVPA). These pedometers include the SC-StepRx (StepsCount Inc, Deep River, ON, Canada), NL-1000 (New-Lifestyles Inc, Lees Summit, MO, USA) and the Walk4Life MVP (Walk4Life, Plainfield, IL, USA). Previous studies have examined the convergent validity of pedometer- and accelerometer-based estimates of MVPA in children and youth [6,7]. However, to our knowledge no study has examined the validity of pedometer-based estimates of MVPA in comparison to the more robust criterion standard of indirect calorimetry. Thus, the purpose of the current study was to determine the validity of the SC-StepRx pedometer to determine MPA, VPA, and MVPA during treadmill walking and running in a heterogeneous sample of children and youth aged 10-17 years. A secondary purpose of this study was to compare the performance of the SC-StepRx with that of the NL-1000 and Walk4Life MVP pedometers, as well as the Actical (Philips Respironics, OR, USA) and ActiGraph GT3X (ActiGraph Corp, Pensacola, FL, USA) accelerometers.

## Methods

### Participants

A convenience sample of 40 participants between the ages of 10 and 17 years participated in this study. Participants under the age of 10 were excluded as it was felt that younger participants would not be able to follow the testing protocol (e.g. maintaining a consistent stepping cadence at various intensities). Informed consent was obtained from participants 16 years of age or older, while parent consent and participant assent were obtained for those under 16 years. Participants were required to complete a health screening questionnaire; be between the ages of 10 and 17 years; and complete at least 4 of the 5 treadmill stages. Research ethics board approval was obtained from the Children’s Hospital of Eastern Ontario Research Institute.

Participants were instructed to abstain from vigorous exercise for 12 hours prior to the testing session, and to avoid all food, drink (except water), caffeine and nicotine for the 2 hours immediately prior to testing. Thirty-two of the 40 participants completed all 5 stages of the treadmill protocol. Four participants exceeded 2 METs at the lowest treadmill speed (0.8 km^.^h^-1^), and therefore did not complete Stage 1. Four participants were unable to maintain the intensity chosen for the final stage (7 METs), and therefore did not complete Stage 5. Participants who did not complete a stage had values set to missing for that particular stage, and were included in all other analyses.

A wall-mounted stadiometer (Seca, Hanover, MD, USA) was used to measure standing and sitting heights to the nearest 0.1 cm, and a digital scale (Seca, Hanover, MD, USA) was used to measure weight to the nearest 0.1 kg for all participants. Leg length was derived from sitting and standing heights. Weight status was determined according to growth charts published by the World Health Organization [8]. Participants who fell more than 1 standard deviation above the growth standard median for their age and gender were considered overweight, and those more than 2 standard deviations above the median were considered obese. Self-rated fitness was assessed by asking participants to indicate on a questionnaire whether their fitness was very good, good, average, poor or very poor. This was based on a similar question in the Canadian Society for Exercise Physiology Physical Activity, Fitness and Lifstyle Approach (CPAFLA) [9] which has not yet been validated. Self-rated fitness has therefore been included only for descriptive purposes.

### Treadmill protocol

**Figure 1:**
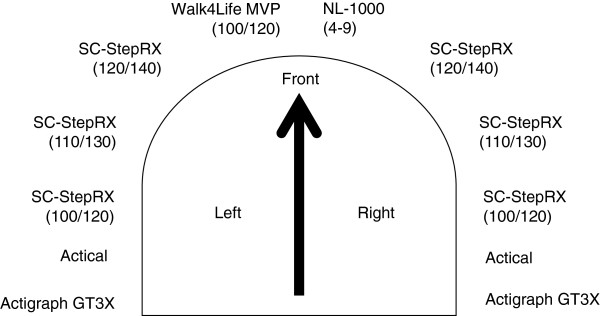
Placement of the physical activity monitors.

The testing protocol was based on a previous study, which examined the validity of the SC-StepMX pedometer to record steps in a heterogeneous sample of children and youth [10]. Participants in the current study wore 6 SC-StepRx pedometers, 2 ActiGraph GT3X accelerometers, 2 Actical accelerometers, 1 NL-1000 pedometer and 1 Walk4Life MVP pedometer (Figure 1). The monitors were evenly spaced on the left and right sides of the body on a belt, and were placed in the same relative positions for all participants.

Participants performed 5 separate 5-minute stages of increasing intensity, with treadmill speeds chosen to approximate 2, 3, 4, 6, and 7 METs, respectively. All stages were performed with a treadmill elevation of 0%. The mean MET value for each stage was determined by averaging energy expenditure during the final 3 minutes of the stage, as determined via indirect calorimetry using a MedGraphics Ultima metabolic cart (MedicalGraphics Corporation, St. Paul, MN, USA). This served as the criterion measure for exercise intensity, with MPA and VPA corresponding to energy expenditures exceeding 4 and 7 METs, respectively [11]. Participants walked or ran on the treadmill for 1–2 minutes prior to each stage in order to determine the treadmill speed approximating the appropriate energy expenditure. Once the appropriate treadmill speed was determined, participants were asked to straddle the treadmill belt while researchers reset all pedometers to zero. Participants then walked or ran at the chosen speed for 5 minutes. Two examiners manually counted and recorded steps during the 3^rd^ and 5^th^ minute of each stage using hand-tally counters. The counts of the two examiners were then averaged, which provided the criterion measure for steps. A video recording was also made of each stage to permit examiners to re-count steps whenever they believed they had made an error in counting. If an examiner believed that an error had taken place, they left the field blank on their scoring sheet during the testing session. After the testing session was complete an examiner used the video to count the steps at the stage with the missing value using the same protocol as above; this new value was then entered into the score sheet to replace the missing value. Across all stages, there were 14 instances when such an error took place. The mean difference between the two examiners was 0.4 steps•minute^−1^, while the greatest absolute difference between examiners at any individual stage was 3 steps•minute^−1^. Across all stages the correlation between the two examiners was R^2^=0.998.

Ratings of perceived exertion [12] were indicated by participants during the 4^th^ minute of each stage. At the conclusion of each stage the number of steps and minutes of MPA and VPA were recorded for each pedometer (the NL-1000 records only total MVPA, and therefore only MVPA was recorded for this activity monitor). Across all time points and monitors, there were 9 specific instances where a pedometer showed evidence that it was not reset prior to a stage (steps > 2 standard deviations from the mean for that stage, and MPA or VPA values which were substantially larger than possible in a 5 minute stage). In these 9 instances the values for the affected pedometer were set to missing for the affected time points.

### Activity monitors & data processing

The current study tested 5 different activity monitors concurrently. This included 3 pedometers (the SC-StepRx, the NL-1000 and the Walk4Life MVP) and 2 accelerometers (The ActiGraph GT3X and the Actical). All 3 pedometers investigated in the current study assess moderate and vigorous physical activity, which is displayed on an LCD screen. The SC-StepRx and the NL-1000 are both piezoelectric pedometers, while the Walk4Life MVP employs a triaxial accelerometer. All three pedometers had similar dimensions. The width, height and depth of the SC-StepRx were 5 cm, 3 cm and 1.5 cm, respectively. The dimensions of the NL-1000 are 6.4 cm × 3.8 cm × 2.2 cm, while those of the Walk4Life MVP are 5.7 cm × 3.2 cm × 1.3 cm. Neither the Actigraph nor Actical have a screen, therefore data from these devices must be downloaded after an activity bout. The ActiGraph accelerometer is a triaxial accelerometer, which has physical dimensions of 3.8 cm × 3.7 cm × 1.8 cm while the Actical is an omnidirectional accelerometer with physical dimensions of 2.8 cm × 2.7 cm × 1.0 cm.

The activity monitors used in the present study allow the user to modify the specific thresholds used to determine MPA and VPA. For pedometers this is done prior to the activity bout, while for accelerometers it is done during data processing. In the current study two SC-StepRx pedometers (one on each side of the body) were set to moderate/vigorous intensity thresholds of 100/120 steps•minute^−1^, two SC-StepRx pedometers were set to moderate/vigorous thresholds of 110/130 steps•minute^−1^, while the final two were set to thresholds of 120/140 steps•minute^−1^. The Walk4Life pedometer was set to moderate/vigorous thresholds of 100/120 steps•minute^−1^, while the NL-1000 pedometer was set to an internal setting of 4–9, which the manufacturer claims is equivalent to 3.6 METs [7]. The settings for all pedometers were confirmed immediately prior to each testing session.

Both Actical and ActiGraph accelerometers were initialized to collect data using 15-second epochs, and collected data on both the number of steps•minute^−1^ and movement intensity. The low frequency extension was used for the ActiGraph accelerometer for all participants [13]. Actical accelerometer cut-points of 1500 and 6500 counts per minute were used to identify MPA and VPA, respectively [14]. ActiGraph data were analyzed using three separate pediatric cut-points; those suggested by Evenson [15], Treuth [16], and Freedson [16,17]. The Evenson cut-points employ thresholds of 2296 and 4012 counts per minute for MPA and VPA, respectively [15], while the Treuth cut-points employ thresholds of 3000 and 5200 counts per minute [16]. Finally, the cut-points developed by Freedson’s group are age-dependent [16,17].

### Statistical analyses

All statistical analyses were performed using SAS 9.2 (SAS Institute Inc, Cary, NC, USA). Data are presented as mean (standard deviation) unless otherwise noted. Mean steps and minutes of MPA, VPA and MVPA were averaged across monitors (within intensity stage) of the same brand and internal settings. The performance of monitors on the left and right sides of the body (within a given brand and intensity stage) were compared using a paired samples *t* test. Baseline differences between male and female participants were assessed using an independent samples *t* test for continuous variables and using the Chi-square test for proportions.

Coefficients of determination (R^2^) were calculated from linear regression analyses to determine the strength of the relationship between the output of each monitor and the criterion measure. With respect to step counts, the criterion measure was manually counted steps. With respect to MPA, VPA and MVPA, the criterion measure was minutes of MPA, VPA and MVPA determined using the metabolic cart. Values of MPA, VPA and MVPA were also dichotomized in order to calculate the sensitivity and specificity of each monitor at each treadmill stage. Using data from the metabolic cart, a stage was considered MPA if the average energy expenditure over the final three minutes of the stage was above 4 METs. Similarly, a stage was considered VPA if the energy expenditure averaged greater than 7 METs. For the activity monitors, a stage was considered MPA if the monitor indicated that ≥ 80% of the stage (e.g. 4 minutes) was spent in MPA. Similarly, a stage was considered VPA if the monitor indicated that ≥ 80% of the stage was spent in VPA. A stage was considered MVPA if the monitor indicated that ≥ 80% of the stage was spent in some combination of MPA and VPA.

Bias was calculated as the difference between the minutes of MVPA recorded via each activity monitor and those recorded via the metabolic cart. Correlation analyses were used to determine whether age, body mass index (BMI), leg length or treadmill speed was associated with bias for each monitor and intensity threshold. Measurement bias was also plotted by treadmill speed.

## Results

Participant characteristics are presented in Table 1. There were no significant differences between male and female participants with respect to age, anthropometric measures, or self-rated fitness. Speed, steps and energy expenditure at each stage are presented in Table 2. The average coefficient of variation for manually counted steps was 0.6%, and ranged from 0 to 5.2%. Across all stages, the lowest speed observed for any individual participant was 0.8 km^.^h^−1^ (Stages 1 and 2), while the highest speed was 8.5 km^.^h^−1^ (Stage 5). The mean (SD) stepping cadence of MPA was 125 (52) steps•min^−1^, while the mean (SD) cadence of VPA was 146 (55) steps•min^−1^. No differences were observed in estimates for steps, MPA or VPA when comparing monitors on the right and left sides of the body (all *p* > 0.05). The ratio of activity monitor step counts to manual step counts are presented in Table 3.

**Table 1:** Participant characteristics

	**Male (n = 20)**	**Female (n = 20)**	***p *** **value**
Age (years)	13.5 (1.5)	13.7 (2.3)	0.55
Height (cm)	161.2 (11.9)	157.9 (11.9)	0.36
Weight (kg)	57.9 (18.8)	53.9 (14.1)	0.45
Leg length (cm)	80.6 (5.8)	81.9 (18.3)	0.76
BMI (kg/m^2^)	21.9 (5.5)	22.3 (3.6)	0.65
Proportion overweight or obese	7/20	5/20	
Overweight	4/20	4/20	1.00
Obese	3/20	1/20	0.29
Self-rated fitness			
Very good	6/20	7/20	0.74
Good	10/20	5/20	0.10
Average	3/20	8/20	0.08
Poor	1/20	0/20	0.31
Very poor	0/20	0/20	1.00

Data are presented as mean (SD). Statistical significance assessed using independent t-tests for continuous variables, and chi-square test for proportions. Overweight and obesity status determined using WHO Growth Charts [8].

**Table 2:** Speed, steps and energy expenditure during each 5-minute stage

						**Steps during stage**					
**Stage**	**Speed in km•h**^**−1**^	**Speed in MPH**	**EE (METs)**	**VO**_**2 **_**(ml•kg**^**−1**^**•min**^**−1**^**)**	**RPE**	**Manual count**	**SC-StepRx**	**Walk4Life**	**NL-1000**	**Actical**	**ActiGraph**
1	1.0 (0.3)	0.6 (0.2)	2.3 (0.3)	8.05 (1.1)	7 (1)	284 (68)	23 (63)	50 (50)	5 (16)	5 (20)	168 (99)
2	2.2 (0.7)	1.4 (0.4)	2.9 (0.2)	10.2 (0.7)	8 (2)	416 (56)	319 (155)	262 (145)	193 (178)	175 (155)	341 (115)
3	3.8 (0.8)	2.4 (0.5)	3.8 (0.3)	13.3 (1.1)	9 (2)	524 (40)	514 (72)	496 (97)	491 (93)	447 (136)	502 (83)
4	5.9 (0.7)	3.7 (0.4)	5.8 (0.5)	20.3 (1.8)	11 (3)	634 (39)	637 (38)	638 (41)	638 (37)	620 (39)	626 (41)
5	6.5 (1.0)	4.1 (0.6)	7.3 (0.7)	25.6 (2.5)	13 (3)	701 (65)	703 (64)	705 (63)	687 (128)	689 (62)	691 (62)

Data are presented as mean (SD). EE: energy expenditure; RPE: rating of perceived exertion.

**Table 3:** Ratio of activity monitor steps to manually counted steps at each stage

**Stage**	**SC-StepRx**	**Walk4Life MVP**	**NL-1000**	**Actical**	**ActiGraph GT3X**
1	8.1	17.6	1.8	1.8	59.2
2	76.7	63.0	46.4	42.1	82.0
3	98.1	94.7	93.7	85.3	97.7
4	100.5	100.6	100.6	97.3	98.7
5	100.3	100.6	98.0	98.3	98.6

Data are presented as percentages, with activity monitor steps as the numerator and manually counted steps as the denominator.

The correlation between manually counted steps and those obtained from activity monitors are presented in Table 4. When all stages were combined, all monitors showed significant associations with manually counted steps, with coefficients of determination ranging from 0.86 (Actical and ActiGraph GT3X) to 0.89 (Walk4Life MVP) (all *p* < 0.05). With the exception of the NL-1000 and Actical during Stage 1 and the Actigraph at Stage 3, steps measured via activity monitors were significantly associated with manually counted steps at all stages, while the observed coefficients of determination tended to increase with treadmill speed for each monitor. There was 1 participant whose pedometer-derived steps at Stage 3 were much lower than for other participants (this was consistent across devices), despite having similar MET values at this stage. When this participant was removed the R^2^ values for Stage 3 increased substantially (e.g. from 0.3 to 0.8) for the SC-StepRx, with more modest increases for the Walk4Life and NL-1000 (e.g. from 0.2 to 0.6, and from 0.3 to 0.5, respectively), with little change for the Actical (e.g. 0.19 to 0.24) and no change for the Actigraph. However, given that the purpose of the present study was to examine the accuracy of activity monitors in a heterogeneous sample, we found no legitimate reason to exclude this participant from the analysis.

**Table 4:** **Coefficients of determination (R**^**2**^**) between each activity monitor and manually counted steps**

**Stage**	**SC-StepRx**	**Walk4Life MVP**	**NL-1000**	**Actical**	**ActiGraph GT3X**
1	0.21*	0.33*	0.05	0.09	0.21*
2	0.35*	0.29*	0.40*	0.30*	0.19*
3	0.37*	0.23*	0.30*	0.19*	0.09
4	0.98*	0.94*	0.97*	0.93*	0.87*
5	0.99*	0.98*	0.99*	0.97*	0.95*
Overall	0.88*	0.89*	0.87*	0.86*	0.86*

*statistically significant association, *p* <0.05.

The correlation between metabolic cart- and activity monitor-determined MPA, VPA and MVPA are shown in Table 5. Significant associations were observed for all monitors and thresholds when examining MVPA, with coefficients of determination ranging from 0.64 (SC-StepRx set to moderate/vigorous thresholds of 100/120 steps•minute^−1^) to 0.82 (NL-1000, and SC-StepRx set to moderate/vigorous thresholds of 110/130 steps•minute^−1^). All associations remained significant when MPA and VPA were analyzed separately, with the exception of the SC-StepRx and Walk4Life MVP pedometers using an MPA threshold of 100 steps•minute^−1^.

**Table 5:** **Coefficients of determination (R**^**2**^**) between each activity monitor and metabolic cart determined MVPA**

**Monitor (Setting)**	**Moderate PA**	**Vigorous PA**	**Moderate and vigorous PA**
SC-StepRX (100, 120)	0.01	0.25*	0.64*
SC-StepRX (110, 130)	0.36*	0.45*	0.82*
SC-StepRX (120, 140)	0.59*	0.51*	0.74*
Walk4Life MVP (100, 120)	0.01	0.25*	0.69*
NL-1000 (4–9)	-	-	0.82*
Actical	0.45*	0.24*	0.78*
ActiGraph GT3X (Evenson)	0.09*	0.21*	0.71*
ActiGraph GT3X (Treuth)	0.29*	0.18*	0.77*
ActiGraph GT3X (Freedson)	0.37*	0.18*	0.81*

*statistically significant association, *p* <0.05.

PA: Physical activity.

The sensitivity and specificity of each monitor for detecting MPA, VPA and MVPA are presented in Table 6. The highest overall sensitivity and specificity for detecting MPA were observed for the Actical accelerometer (Sensitivity: 80.3; Specificity: 84.1). The highest overall sensitivity and specificity for both VPA (Sensitivity: 100.0; Specificity: 88.6) and MVPA (Sensitivity: 92.9; Specificity: 96.5) were observed for the SC-StepRx set to moderate/vigorous thresholds of 110/130 steps•minute^−1^.

**Table 6:** Sensitivity and specificity of each activity monitor for moderate and vigorous physical activity

**Monitor**	**Moderate PA**		**Vigorous PA**		**Moderate and Vigorous PA**	
	**Sensitivity**	**Specificity**	**Sensitivity**	**Specificity**	**Sensitivity**	**Specificity**
SC-StepRx (100, 120)	16.4	89.9	100.0	74.3	98.8	84.2
SC-StepRx (110, 130)	44.3	97.1	100.0	88.6	92.9	96.5
SC-StepRx (120, 140)	27.9	98.6	45.8	98.9	77.6	99.1
Walk4Life MVP (100, 120)	16.4	91.3	95.8	77.1	97.7	87.7
NL-1000 (4–9)	-	-	-	-	85.9	97.4
Actical	80.3	84.1	37.5	97.1	91.8	91.2
ActiGraph GT3X (Evenson)	26.2	92.0	70.8	82.9	87.1	92.1
ActiGraph GT3X (Treuth)	39.3	92.0	41.7	92.6	76.5	98.3
ActiGraph GT3X (Freedson)	62.3	89.1	37.5	94.9	87.1	96.5

PA: Physical activity.

**Figure 2:**
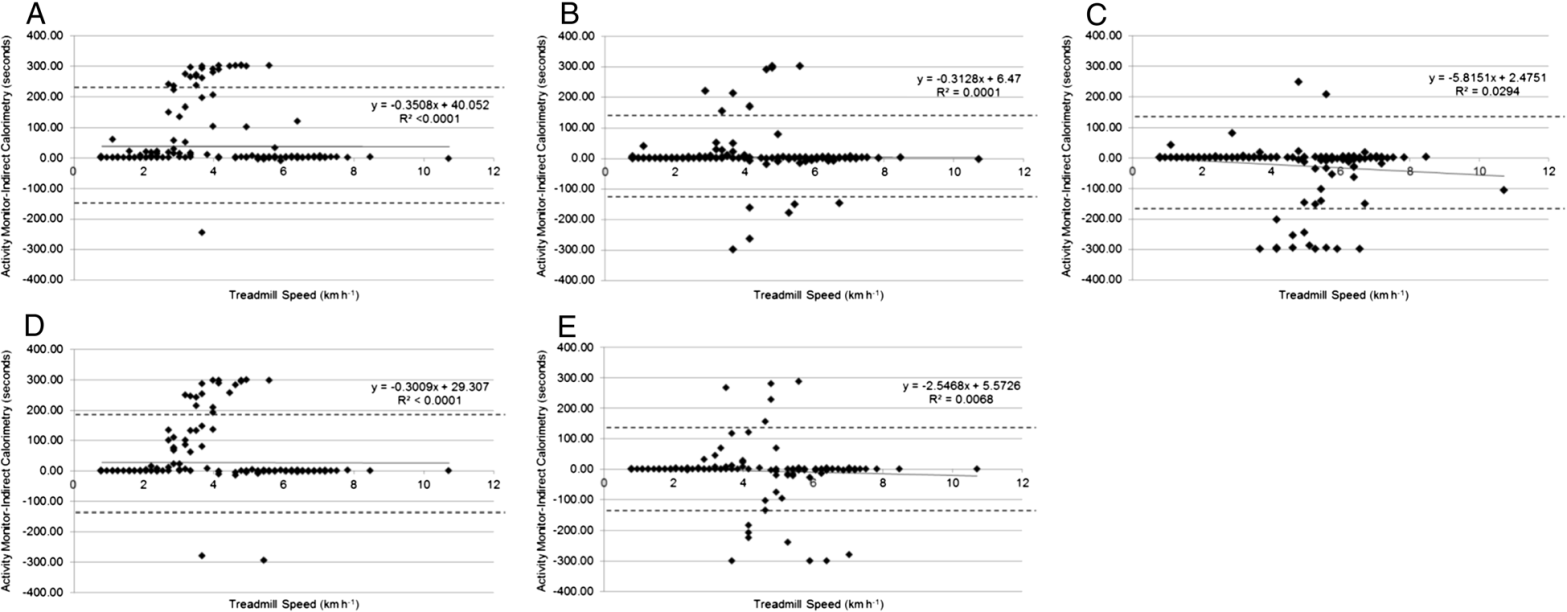
**Bias at different treadmill speeds in SC-StepRx, Walk4Life MVP and NL-1000. A**: SC-StepRx with moderate/vigorous thresholds of 100/120 steps•minute^−1^; **B**: SC-StepRx with moderate/vigorous thresholds of 110/130 steps•minute^−1^; **C**: SC-StepRx with moderate/vigorous thresholds of 120/140 steps•minute^−1^; **D**: Walk4Life MVP with moderate/vigorous thresholds of 100/120 steps•minute^−1^; **E**: NL-1000 pedometer with an internal setting of 4–9 (no units).

**Figure 3:**
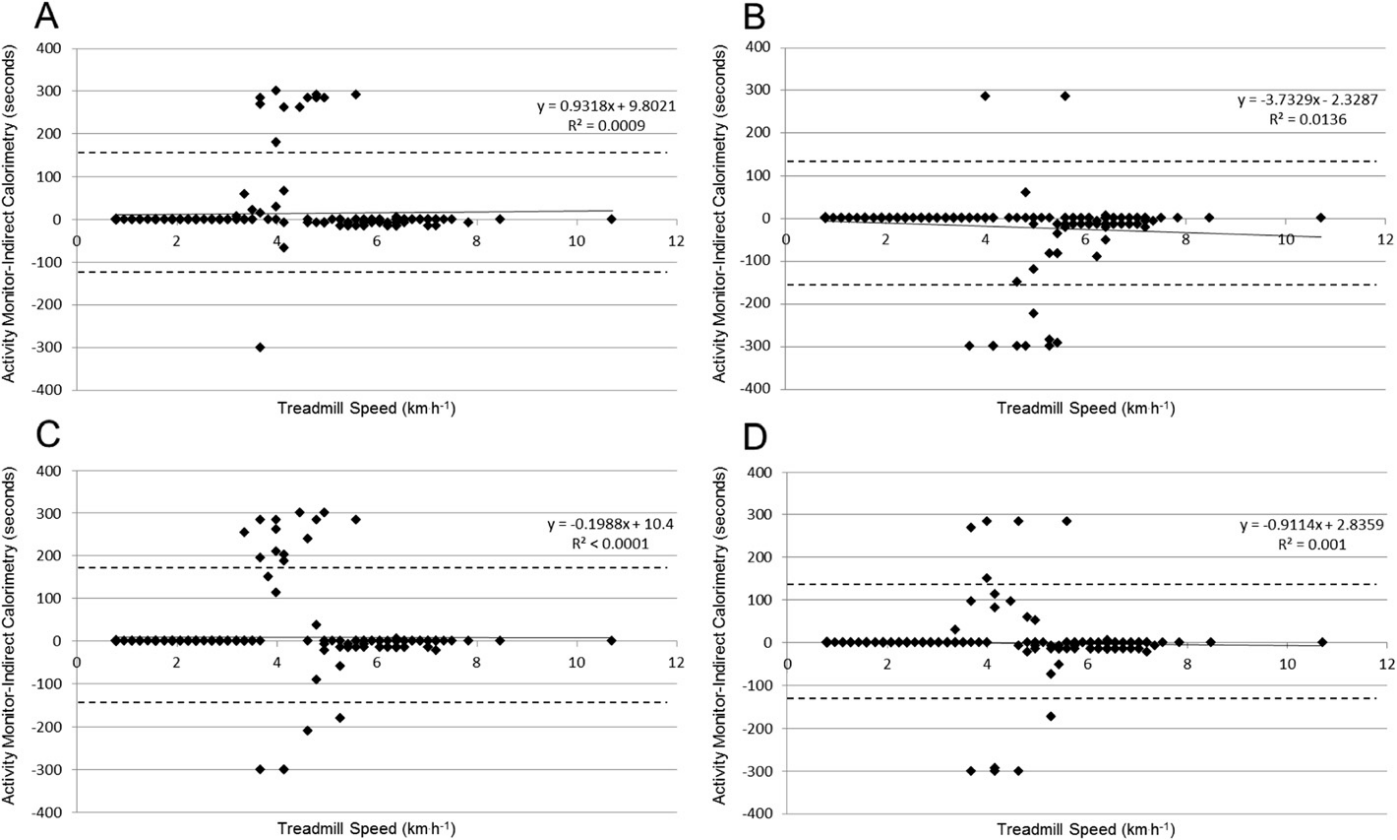
**Bias at different treadmill speeds in the Actical and ActiGraph GT3X accelerometer. A**: Actical accelerometer; **B**: ActiGraph GT3X using Treuth cut-points; **C**: ActiGraph GT3X using Evenson cut-points; **D**: ActiGraph GT3X using Freedson cut-points.

The bias results for MVPA relative to treadmill speed are presented for pedometers and accelerometers in Figures 2 and 3, respectively. Across all monitors, the most narrow limits of agreement were seen for the SC-StepRx with moderate/vigorous thresholds of 110/130 steps•minute^−1^. In correlation analyses bias was not associated with age, BMI, weight status, leg length or treadmill speed when examining SC-StepRx pedometers set to moderate/vigorous thresholds of 100/120 steps•minute^−1^ or 110/130 steps•minute^−1^, NL-1000 pedometers, or ActiGraph GT3X accelerometers using Evenson or Freedson cut-points (all p >0.05). When examining SC-StepRx pedometers set to moderate/vigorous thresholds of 120/140 steps•minute^−1^, bias was negatively associated with treadmill speed only (r = −0.171, p = 0.02). Bias of the Walk4Life MVP with moderate/vigorous thresholds of 100/120 steps•minute^−1^ was positively associated with BMI only (r = 0.145, p = 0.04). Bias of the Actical accelerometer was associated with BMI (r = 0.171, p = 0.02) and weight status (0.162, p = 0.03). Using the Treuth cut-points, bias of the ActiGraph GT3X was associated with age only (r = 0.168, p = 0.02).

## Discussion

The results of the present study suggest that the SC-StepRx demonstrates validity for the measurement of MPA, VPA and MVPA compared to values determined via indirect calorimetry. When set to moderate/vigorous thresholds of 110/130 steps•min^−1^, the association between SC-StepRx and metabolic cart-determined MPA, VPA and MVPA were comparable or greater than those observed for other pedometers or accelerometers. Similarly, using moderate/vigorous thresholds of 110/130 steps•min^−1^, the SC-StepRx had the highest combined sensitivity and specificity for detecting MVPA and the narrowest limits of agreement of any monitor in the current study.

To our knowledge, this is the first study to directly compare values of MPA, VPA and MVPA determined via pedometers with those obtained via indirect calorimetry. Previous studies have instead compared MVPA values determined via pedometer with those determined via accelerometry [6,7]. The current findings illustrate the potential drawbacks to such an approach. For example, Duncan et al. [7] have recently reported that the NL-1000 pedometer showed a low level of precision for MVPA, when compared to the Actical accelerometer in a group of children aged 5–11 years. In contrast, in the present study the NL-1000 and Actical displayed comparable levels of association, sensitivity, specificity, and limits of agreement with the criterion measure of indirect calorimetry.

In the current study, we observed relatively small associations between bias and age, BMI, leg length, and treadmill speed for all activity monitors. It is not surprising that bias for all monitors tended to cluster around the treadmill speeds associated with exercise intensities just above and below the threshold for MVPA. It is possible that this bias could be reduced by determining the stepping cadence associated with MVPA for individual participants, although such an approach may be overly onerous for most studies. Further, the results of the present study suggest that moderate/vigorous thresholds of 110/130 steps•minute^−1^ are able to minimize bias when using the SC-StepRx to assess MVPA in the pediatric age group.

The current findings build on our previous study [10], which found that the association between activity monitor and manually counted steps increased with walking speed in a group of participants ranging in age from 12–58 years. In the current study, the association between activity monitor and manually counted steps was relatively weak (R^2^ ≤ 0.40) for all monitors during the first 3 treadmill stages, but increased dramatically (R^2^ ≥ 0.87) for all monitors at stages 4 and 5. When combined with the results of our previous study [10], these findings also suggest that the ability of activity monitors to accurately assess steps at slow speeds may be lower in children and youth than in adults. For example, the association between activity monitor and manually counted steps during stage 3 (mean speed = 3.8 km^.^h^−1^) of the present study displayed a coefficient of variation of 0.37 for the SC-StepRx and 0.19 for the Actical. In contrast, similar models displayed coefficients of variation of 0.83 and 0.59, respectively, at the same average speed in our previous study, which included predominantly adult participants [10]. It is therefore likely that at slow walking speeds, step count values determined using activity monitors in children may be less accurate than those determined in adults.

Current evidence suggests that 100 steps•minute-^1^ represents a useful threshold for MPA in adults [18]. Manual count data from the present study suggests that the transition from light to moderate PA likely occurs between 100 and 110 steps•minute-^1^ in children and youth. In support of this, we found that an MPA threshold of 110 steps•minute-^1^ had higher sensitivity and specificity using the SC-StepRx than thresholds of 100 or 120 steps•minute-^1^. However, it is important to note that the current investigation employed MPA and VPA thresholds of 4 and 7 METs, respectively, which are lower than the MPA and VPA thresholds of 3 and 6 METs typically used for adults [11]. These higher thresholds have been suggested for use in children and youth in order to account for their higher level of resting energy expenditure, which decrease with both age and maturation [11,19,20]. For example, Harrell et al. [20] have recently reported that oxygen uptake at rest declines from 6.08 ml•kg^−1^•min^−1^ at Tanner Stage 1 to 3.80 ml•kg^−1^•min^−1^at Tanner Stage 5. Employing thresholds of 3 and 6 METs rather than 4 and 7 METs may have resulted in lower steps•minute-^1^ thresholds closer to those observed in adults (e.g. 100 steps•minute-^1^).

The current study has several strengths and limitations that warrant mention. Although several different MPA and VPA thresholds were examined for the SC-StepRx, only one set of thresholds was examined for the Walk4Life MVP and the NL-1000. Given that the performance of the Walk4Life MVP with moderate/vigorous thresholds of 100/120 steps•minute^−1^ was very similar to that observed for the SC-StepRx at the same setting, it is possible that the Walk4Life would show similar performance at other thresholds as well. Further investigations comparing the SC-StepRx and Walk4Life MVP at various moderate/vigorous thresholds to indirect calorimetry are therefore warranted. It should also be noted that the current results may have been influenced by the location of each individual activity monitor. The monitors were placed in the same locations for all participants, meaning that the location of the SC-StepRx was closer to the recommended placement than that of the other monitors. However, previous studies suggest that pedometer placement has little or no significant impact on step counts in children and youth [21-23]. Future studies comparing pedometers should nonetheless consider randomizing the placement of activity monitors in order to minimize any impact of placement. The current study used a relatively small, lab-based design, with participants walking at prescribed speeds and intensities, which allowed us to compare the performance of multiple monitors to a criterion standard at a wide range of exercise intensities. Other studies are needed to validate the SC-StepRx in larger samples using a more naturalistic design. The results of the present study are strengthened by the inclusion of a wide range of participant ages (10–17 years) and body sizes.

## Conclusions

The present findings suggest that the SC-StepRx pedometer is a valid tool for the assessment of MPA, VPA, and MVPA in the pediatric age group. When employing moderate/vigorous thresholds of 110/130 steps•minute^−1^, the performance of the SC-StepRx was comparable or superior to that observed from other pedometers and accelerometers. These results suggest that the SC-StepRx may serve as an inexpensive tool for the assessment of MVPA in clinical and research contexts in children and youth.

## Competing interests

This study was funded by StepsCount Inc., Deep River, Ontario. The funder had no role in the data collection, analysis or the preparation of this manuscript. The authors declare that they have no other competing interests.

## Authors’ contributions

TJS and MST assisted with the design of the study. TJS, CEG, MMB, A McFarlane, A Mbonu, and ZMF assisted with data collection. TJS led the statistical analysis, with input from CEG, MMB and MST. TJS wrote the initial draft of the article, and received important intellectual content from CEG, MMB, A McFarlane, A Mbonu, ZMF and MST. All authors approve the version that has been submitted.

## Pre-publication history

The pre-publication history for this paper can be accessed here:

http://www.biomedcentral.com/1471-2458/14/519/prepub

## References

[B1] Tremblay MS Warburton DER Janssen I Paterson DH Latimer AE Rhodes RE Kho ME Hicks A LeBlanc AG Zehr L Murumets K Duggan M New Canadian physical activity guidelines Appl Physiol Nutr Metab 2011 36 36 46 10.1139/H11-009 21326376

[B2] Janssen I LeBlanc AG Systematic review of the health benefits of physical activity and fitness in school-aged children and youth Int J Behav Nutr Phys Act 2010 7 40 10.1186/1479-5868-7-40 20459784 PMC2885312

[B3] Beets MW Bornstein D Beighle A Cardinal BJ Morgan CF Pedometer-measured physical activity patterns of youth: a 13-country review Am J Prev Med 2010 38 208 216 10.1016/j.amepre.2009.09.045 20117578

[B4] Clemes SA Biddle SJH The use of pedometers for monitoring physical activity in children and adolescents: measurement considerations J Phys Act Health 2013 10 249 262 23407447 10.1123/jpah.10.2.249

[B5] Craig CL Cameron C Tudor-Locke C CANPLAY pedometer normative reference data for 21,271 children and 12,956 adolescents Med Sci Sports Exerc 2013 45 123 129 10.1249/MSS.0b013e31826a0f3a 22843106

[B6] Beets MW Morgan CF Banda JA Bornstein D Byun W Mitchell J Munselle L Rooney L Beighle A Erwin H Convergent validity of pedometer and accelerometer estimates of moderate-to-vigorous physical activity of youth J Phys Act Health 2011 8 Suppl 2 S295 S305 28829707 10.1123/jpah.8.s2.s295

[B7] Duncan S White K Sa’ulilo L Schofield G Convergent validity of a piezoelectric pedometer and an omnidirectional accelerometer for measuring children’s physical activity Pediatr Exerc Sci 2011 23 399 410 21881160 10.1123/pes.23.3.399

[B8] The WHO Child Growth Standardshttp://www.who.int/childgrowth/en/

[B9] Canadian Society for Exercise Physiology (CSEP) The Canadian Physical Activity, Fitness and Lifestyle Approach (CPAFLA) 2003 3 Ottawa: Canadian Society for Exercise Physiology

[B10] Colley RC Barnes JD Leblanc AG Borghese M Boyer C Tremblay MS Validity of the SC-StepMX pedometer during treadmill walking and running Appl Physiol Nutr Metab 2013 38 520 524 10.1139/apnm-2012-0321 23668759

[B11] Troiano RP Berrigan D Dodd KW Mâsse LC Tilert T McDowell M Physical activity in the United States measured by accelerometer Med Sci Sports Exerc 2008 40 181 10.1249/mss.0b013e31815a51b3 18091006

[B12] Borg GA Psychophysical bases of perceived exertion Med Sci Sports Exerc 1982 14 377 381 7154893

[B13] Cain KL Conway TL Adams MA Husak LE Sallis JF Comparison of older and newer generations of ActiGraph accelerometers with the normal filter and the low frequency extension Int J Behav Nutr Phys Act 2013 10 51 10.1186/1479-5868-10-51 23618461 PMC3641979

[B14] Puyau MR Adolph AL Vohra FA Zakeri I Butte NF Prediction of activity energy expenditure using accelerometers in children Med Sci Sports Exerc 2004 36 1625 1631 15354047

[B15] Evenson KR Catellier DJ Gill K Ondrak KS McMurray RG Calibration of two objective measures of physical activity for children J Sports Sci 2008 26 1557 1565 10.1080/02640410802334196 18949660

[B16] Treuth MS Schmitz KH Catellier D McMurray RG Murray D Almeida MJ Going S Norman JE Pate R Defining accelerometer thresholds for activity intensities in adolescent girls Med Sci Sports Exerc 2004 36 1259 1266 15235335 PMC2423321

[B17] Trost SG Pate RR Sallis JF Freedson PS Taylor WC Dowda M Sirard J Age and gender differences in objectively measured physical activity in youth Med Sci Sports Exerc 2002 34 350 355 10.1097/00005768-200202000-00025 11828247

[B18] Tudor-Locke C Craig CL Brown WJ Clemes SA De Cocker K Giles-Corti B Hatano Y Inoue S Matsudo SM Mutrie N Oppert JM Rowe DA Schmidt MD Schofield GM Spence JC Teixeira PJ Tully MA Blair SN How many steps/day are enough? For adults Int J Behav Nutr Phys Act 2013 8 79 21798015 10.1186/1479-5868-8-79 PMC3197470

[B19] Cain KL Sallis JF Conway TL Van Dyck D Calhoon L Using accelerometers in youth physical activity studies: a review of methods J Phys Act Health 2013 10 437 450 23620392 10.1123/jpah.10.3.437 PMC6331211

[B20] Harrell JS McMurray RG Baggett CD Pennell ML Pearce PF Bangdiwala SI Energy costs of physical activities in children and adolescents Med Sci Sports Exerc 2005 37 329 336 10.1249/01.MSS.0000153115.33762.3F 15692331

[B21] Abel MG Hannon JC Eisenman PA Ransdell LB Pett M Williams DP Waist circumference, pedometer placement, and step-counting accuracy in youth Res Q Exerc Sport 2009 80 434 444 10.1080/02701367.2009.10599581 19791629

[B22] Graser SV Pangrazi RP Vincent WJ Effects of placement, attachment, and weight classification on pedometer accuracy J Phys Act Health 2007 4 359 369 18209228 10.1123/jpah.4.4.359

[B23] Jago R Watson K Baranowski T Zakeri I Yoo S Baranowski J Conry K Pedometer reliability, validity and daily activity targets among 10- to 15-year-old boys J Sports Sci 2006 24 241 251 10.1080/02640410500141661 16368634

